# Roles of Microglia in Synaptogenesis, Synaptic Pruning, and Synaptic Plasticity in Physiological Conditions and Central Nervous System Disorders

**DOI:** 10.2174/1570159X23666250225091729

**Published:** 2025-02-26

**Authors:** Meizhen Xie, Tian Wang, Jiachun Feng, Di Ma, Liangshu Feng, Yulei Hao

**Affiliations:** 1 Cell Biology, Neurobiology and Biophysics, Department of Biology, Faculty of Science, Utrecht University, Utrecht 3584 CH, The Netherlands;; 2 The Second Hospital of Jilin University, Changchun, Jilin Province 130021, People’s Republic of China;; 3 Department of Neurology and Neuroscience Center, The First Hospital of Jilin University, Xinmin Street No. 1, Changchun, Jilin Province 130021, China;; 4 Stroke Center, Department of Neurology, The First Hospital of Jilin University, Xinmin Street No. 1, Changchun, Jilin Province 130021, China;; 5 Doctor of Excellence Program (DEP), The First Hospital of Jilin University, Xinmin Street No. 1, Changchun, Jilin Province 130021, China

**Keywords:** microglia, synapse, synaptogenesis, synaptic plasticity, Alzheimer’s disease, Parkinson’s disease, transcriptomic, ischemic stroke

## Abstract

Microglia are resident immune cells in the brain that have been widely studied for their immune surveillance and phagocytosis. In recent years, the important role of microglia in synapse formation, elimination, and plasticity is gradually being recognized. Synapses are the main communication mode between neurons. They undergo constant changes in quantity and plasticity throughout the life cycle, which is the basis of learning and memory. Microglia are highly motile, branched forms that monitor the microenvironment of the central nervous system (CNS) and promote synapse formation and maturation. They recognize and phagocytose redundant synapses through specific phagocytosis receptors. Furthermore, microglia regulate synaptic plasticity by releasing various effectors. The roles of microglia on synapses ensure the proper function of neural networks. Synaptic dysfunction and microglia activation are common features in CNS disorders, such as Alzheimer's disease, Parkinson's disease, ischemic stroke, cerebral hemorrhage, traumatic brain injury, multiple sclerosis, and epilepsy. Highly heterogeneous microglia exhibit diverse functions in these diseases and participate in disease progression by exacerbating or inhibiting synaptic dysfunction, in addition to neuroimmune and inflammation. In this article, we summarize the role of microglia on synapses under physiological conditions and in CNS disorders. We highlight the possible mechanisms by which microglia regulate synapse function in CNS disorders and how this affects the progression of the diseases. We aim to explore potential therapeutic targets for CNS disorders.

## 1 INTRODUCTION

Microglia are resident brain cells that account for approximately 10% of cells in the central nervous system (CNS) [1, 2]. And microglia are highly heterogeneous in morphology and function due to their high transcriptional plasticity [3]. In physiological conditions, they may operate as structural cells to provide nutritional and structural support for the development of neurons and synapses and as immune cells with macrophage-like roles to monitor and clear excess or abnormal synapses and influence synaptic plasticity by releasing cytokines [4]. Therefore, microglia are essential for the maintenance of normal neural circuits. Synaptic dysfunction is an important pathological feature and pathogenesis of many CNS disorders, including neurodegenerative diseases, epilepsy, and cerebrovascular disease. There are many causes of synaptic dysfunction, including abnormal deposition of pathological proteins, imbalance of neurotransmitter transmission, and stimulation resulting from cerebral ischemia and hypoxia [5-7]. In contrast, as one of the major cells regulating synaptic function in physiological states, the effect of microglia on synaptic function in CNS disease states deserves attention. Indeed, microglia rapidly respond and exhibit different states and functions in CNS diseases. For example, microglia can aggravate synaptic dysfunction by releasing inflammatory factors [8], as well as reduce neural tissue damage by phagocytosis of dead or damaged synaptic fragments [9]. They can also promote synaptogenesis and remodeling by releasing neurotrophic factors [10]. Therefore, targeting microglia in a specific state may make it possible to achieve rescue of synaptic damage and promote neural function recovery. Past concepts defined microglia simply as the “M1” and “M2” phenotypes [11]. Studies targeting these two phenotypes have been proven to be effective in different diseases [12-14]. However, it was gradually recognized that this classification was too limited to fully explain the heterogeneity of microglia. The application of single-cell sequencing has provided new insights into microglia heterogeneity [3]. In this paper, we summarize the effects of microglia on synaptic function in physiological conditions and CNS disorders and analyze the diverse effects of highly heterogeneous microglia on synapses in different diseases. Finally, we propose that the application of single-cell sequencing in CNS diseases provides a viable option for future studies on targeting microglia precisely.

## 2 MICROGLIA

### 2.1 The Origin and Development of Microglia in the Central Nervous System

Microglia were first discovered and described a century ago (1919). It was initially thought that microglia originated from the neuroectoderm, like that of most cells in the CNS. However, later work found that microglia originate from the erythromyeloid progenitor cells (EMPs) of the yolk sac of the mesoderm. Fate-mapping studies in mice have shown microglia have the same origin as tissue-resident macrophages [15], and therefore, microglia are known as CNS macrophages [16]. In mice, differentiation from EMPs to microglial progenitors begins on embryonic day 8 (E8). Microglial progenitors then move closer to the embryonic brain. These progenitors surround the neuroepithelium at around E9.5 and gradually enter the neuroepithelium after one day [17, 18]. Migration may continue until blood-brain barrier (BBB) development is complete. Thereafter, microglia remain in the nervous system for life and gradually develop into mature microglia.

The number of microglia that migrate into the CNS expands from pre-birth to the early period after birth. In mice, the number of microglia significantly increases two times before birth. The first increasing phase occurs between E8.5 and E14.5 when microglia progenitors colonize the brain, accompanied by the rapid proliferation of microglia that had entered the brain. The researchers discovered that microglia rely on the circulatory system to get to the brain because they are not found in the brains of embryos without blood circulation [17, 19]. The second increasing phase of microglia number occurs between E14 and E16, which is the second invasion process of microglia precursor cells. The number of microglia continues to increase at a slow rate until E17.5 and continuously self-renew to maintain their numbers [20]. Microglia numbers also increase within the first two weeks after birth and begin to decrease in the fourth week; this may be related to microglia apoptosis and tends to stabilize in the sixth week [21]. During human embryonic development, microglia first appear at 5.5 gestational weeks (gw) near the di-telencephalic fissure and choroid plexus. Between 9 and 14 gw, microglia proliferate rapidly, and after 16 gw, large numbers of microglia are distributed throughout the brain parenchyma [22].

Microglia cannot be renewed by peripheral bone marrow progenitor cells under physiological conditions, unlike peripheral monocytes. However, they can slowly proliferate in the local cell pool of the brain, which is the only regeneration site of microglia in the CNS [23]. Microglia have a long life span (up to 4.2 years in the human brain) and renew very slowly in mammals [24]. Aging microglia are mainly eliminated through apoptosis, and new microglia proliferate in the cell pool; therefore, microglia are relatively “resting cells”.

The genesis and development of microglia are tightly regulated and require the coordination of multiple molecules, including but not limited to transcription factors, growth factors, and microRNAs. Microglia are strongly dependent on continuous stimulation of the colony-stimulating factor 1 receptor (CSF1R) [25]. The CSF1R signaling pathway can increase the nutrition of microglia, inhibit the proliferation of forebrain progenitors [26], promote differentiation, and ensure the survival of neural progenitors and precursor cells [27]. This ensures microglial survival and maintains their physiological function. In addition, TGF-β has been proven to be the regulator of microglia differentiation both *in vitro* and *in vivo*. Mice lacking TGF-β show the loss of microglia in the CNS along with defects in glutamate homeostasis and synaptic plasticity [28]. Astrocytes and neurons can also regulate microglial homeostasis by providing immune signals in a TGF-β-dependent manner that inhibit microglial responses to weaker inflammatory stimuli [25]. Furthermore, several transcription factors, including SPI1 (PU.1), interferon regulatory factor 8 (IRF8), Sall1, and Mafb, are essential for microglia development [16, 29, 30]. PU.1 and IRF-8 can promote the differentiation of microglia precursors and EMPs in the yolk sac [31]. Spi1 (PU.1) is the most critical transcription factor, and it is the most abundantly expressed ETS domain transcription factor in human microglia [32]. The expression level of PU.1 can directly affect the function of microglia, and it can also affect the expression of basic survival receptors such as CSF1R in microglia, hence precisely maintaining the homeostasis and function of microglia. PU.1 contains the family binding motifs of Smad, Mef2, and Ctcfl. These transcription factors cooperate with PU.1 to establish the microglia-specific gene enhancer profiles [16]. In addition, IRF8 can maintain the homeostasis of mature microglia and control the activation of microglia. IRF8 deficiency in adult mice results in an increased abundance of microglia accompanied by microglia reaction phenotype [33]. Sall1 is a zinc-finger transcription factor and is expressed in the pre-microglia stage. It is a key marker of microglia and a regulator of the normal development of microglia in the embryonic brain [34]. Scott *et al.* found that the macrophage mannose receptor CD206 in normal microglia is down-regulated at the early embryonic stage (E13.5), while the knockout of Sall1 in mice resulted in sustained expression of CD206 in microglia and reduced morphological branching of microglia, CD206 is a marker of microglia activation, and these results suggest that SALL1 plays a role in the repression of CD206 and the maintenance of microglia morphology both in an early development stage and after birth [35]. Mabf is believed to be involved in the regulation of microglia homeostasis in adults. Specific knockdown of Mafb in microglia results in enhanced expression of interferon and inflammatory pathways, ultimately leading to immune imbalance in microglia [23]. MicroRNA (miR) may also be a regulator of microglial development. It has also been found that brain-specific miR-124 is highly expressed in microglia and proved that it can maintain the quiescent state of microglia, inhibit the proliferation of microglia, and induce the differentiation of microglia precursors into mature microglia. In addition, miR-124 was shown to be an upstream signal of PU.1, which could regulate microglia by down-regulating PU.1 expression [36].

### 2.2 The Distribution and Morphology of Microglia

Microglia are diverse in spatial distribution and cell morphology in the CNS. In terms of spatial distribution, microglia are mainly located in the gray matter, and the highest distribution density of microglia is in the hippocampus, followed by the striatum and cortex (at slightly lower levels than that in the hippocampus), and the thalamus and midbrain. Meanwhile, microglia levels in the cerebellum are low, and the density of microglia in the retina and spinal cord is similar to that in the midbrain or cerebellum [37, 38]. Thus, the distribution of microglia follows the rule of decreasing density from rostral to caudal- and from dorsal to ventral directions [38].

In terms of cell morphology, microglia exist as unbranched amoeba-like from the differentiation of bone marrow progenitor cells until colonization in the CNS and gradually become branched after colonization in the CNS until completion on the 28^th^ day after birth [17]. The degree of microglia branching differs and is related to the distribution and cell status. For example, the number of microglial branches in the hippocampus and cortex is much denser than that in the cerebellum [38]. Stress conditions such as inflammation and trauma can change branched microglia to amoeboid morphology [37]. The migration ability of microglia is enhanced after stress and targeted movement to the injured site. Chemotaxis depends on the binding of activated P2Y12 receptors on microglia to ATP and ADP released by neural cells [39, 40]. *In vivo* and *in vitro* studies showed that the mobility of microglia peaks at approximately 3 days after brain injury, and returns to the pre-injury level after 5 days [21]. Microglia are not permanently in contact with each other, their spatial distribution is significantly “repulsive”; however, the distance between them decreases with increasing cell density, which may be related to the intensity of neural activity [41].

### 2.3 Microglia Heterogeneity

Traditionally, it is thought that in physiological conditions, microglia are in a low-phagocytic branching form called resting microglia (M0) [42]. There are two main functional phenotypes of microglia after disease: pro-inflammatory M1 and anti-inflammatory M2. The M1 phenotype is activated under the stimulus of acute inflammation and induces the release of pro-inflammatory factors, chemokines, and cytotoxic substances, which ultimately triggers pro-inflammatory effects. The M2 phenotype plays an anti-inflammatory role under the stimulation of chronic inflammation by releasing cytokines, including IL-10 and extracellular matrix (ECM) proteins; this helps protect cells and tissues [43]. M1 and M2 phenotypes are different activation states of microglia in response to external inflammatory signals, rather than the final differentiation state of microglia. Microglia can express both M1 and M2 markers at the same time, and the two phenotypes can be interconverted [44].

With further study of microglia, it is found that the dichotomous classification (such as M1, M2; resting, activated) has rather limited the potential role of microglia. Microglia are always active in adapting their state to their surroundings, whether in physiological or pathological conditions. To specifically describe the functional states of microglia in different, dynamic, multidimensional environments, researchers have recently named microglia in specific contexts based on single-cell technology, multi-omics gene, and protein expression analysis. The heterogeneity of microglia depends on these contexts, including age, CNS regions, circadian time, gender, and CNS diseases [3].

Microglia exhibit region-dependent diversity in the central nervous system. The researchers isolated microglia from different brain regions after human death, using extensive single-cell immune profiling. They identified four microglia clusters that showed differential abundance in different brain regions, and cluster1 was specifically present in the thalamus and subventricular zones but not in other brain regions. They also expressed more CD11c, CD195 (CCR5), CD45, CD64, CD68, CX3CR1, EMR1 and HLA-DR than microglia in other brain regions. Cluster 2 and 3 were more abundant in the temporal lobe and frontal lobe, while cluster 4 was rare [45].

Microglia also show transcriptional differences between males and females. For example, genes involved in the type I interferon pathway, including Ifnb1, CCRL2, and Cxcl10, are more expressed in microglia of females, which are involved in the immune system and may be related to diseases, such as the greater susceptibility of women to autoimmune diseases [46]. In addition, the expression of diurnal clock genes is suppressed in the microglia of the aged compared with the young, and the disruption of circadian rhythm is associated with aging and degenerative diseases [47]. Homeostatic microglia (H1Ms and H2Ms) have also been identified, which show significant gene expression differences in different brain regions, genders, and ages [48]. Many different microglial states have also been found in disease species and models, including disease-associated microglia (DAMs) [49], microglial neurodegenerative phenotype (MGnD) [50], activated response microglia (ARMs) [51], interferon-responsive microglia (IRMs) [52], human Alzheimer’s disease (AD) microglia (HAMs), human HIV-infected microglia [53], PD-associated microglia [54], microglia inflamed in MS (MIMS) [55] *etc*., which we will elaborate in CNS diseases in part 4.

In conclusion, microglia exhibit heterogeneity in cellular state and temporal and spatial localization, which helps them to play a more precise role in the CNS. Furthermore, microglial activation, inflammation, and disease progression can be better tracked and visualized by identifying reliable markers of different microglial states.

### 2.4 Microglia Functions

Microglia are multifunctional cells that play many roles in the CNS, including immune surveillance, phagocytosis, synaptic pruning, and trophic roles.

#### 2.4.1 Immune Surveillance

Microglia are the most important innate immune cells in the CNS and play a continuous role in immune surveillance under physiological conditions. The rapid retractable characteristic of the microglia branch is proved despite the slow rate of proliferation and almost no movement of the cell body, which favors microglia to receive signals from other cells [39, 56]. Microglial receptors such as CD200R and CX3CR1 can receive messages from astrocytes and oligodendrocytes, and lipid mediators secreted by microglia such as anandamide in the homeostatic brain; this helps microglia stay in a non-reactive state [43]. However, non-reactive microglia are not really “resting”. When the surrounding environment is threatened and disturbed, microglia act as “first responders” by cooperating with other glial cells to immediately adjust the activity of neurons [57].

#### 2.4.2 Phagocytosis

Microglia receive signals from non-self-immunogens such as bacteria, fungi, viruses, or self-immunogens, including the intracellular constituents released from necrotic or apoptotic cells, and rapidly activate to produce a series of responses. The state of microglia changes to accommodate an increase in migration ability [21], the expression of pro-inflammatory factors (IL-1α, TNF-α), chemokines, and cytotoxic substances. In addition, microglia can act as antigen-presenting cells, presenting antigens in major histocompatibility complex I (MHC I) and MHC II complexes to CD8^+^ and CD4^+^ cells. Activated T cells can stimulate microglia through the secretion of TNF-α, thereby regulating the immune function of microglia [17, 25]. As the disease progresses, microglia play a protective role by releasing anti-inflammatory factors and participating in the phagocytosis of necrotic cells to mediate immune repair [43].

Phagocytosis is also essential for microglia to participate in the homeostasis regulation of the nervous system. This process is reflected throughout the life cycle and occurs in brain development, disease, aging, and in inflammatory- or non-inflammatory environments [56]. The effect of phagocytosis has dual roles. Phagocytosis of aging neurons or cell debris by microglia helps reduce inflammation and avoid more brain damage under physiological conditions. At the same time, faulty or excessive phagocytosis of microglia can promote the progression of the disease in pathological conditions [58]. Notably, phagocytosis of microglia also plays an important role in synaptic pruning, which will be elaborated in Section 3.

#### 2.4.3 Trophic Roles

Although astrocytes are considered the energy pool of the brain, microglia can also secrete nutritional factors such as BDNF that provide important support for the proper functioning of the brain. These trophic factors contribute to neuronal survival and synapse formation during brain development. In adulthood, microglia provide trophic factors that support neuronal activity and contribute to the formation of learning-dependent synapses in the motor cortex [17, 30]. The nutritional support provided by microglia reduces the extent of brain damage during slight disruptions to the homeostasis of the nervous system; therefore, it is essential for brain protection [43].

#### 2.4.4 Effect on Perineuronal Networks

Pioneering studies in recent years have shown that microglia can play a role in regulating homeostasis as well by controlling the remodeling of perineuronal networks (PNNs), which are a kind of specialized extracellular matrix (ECM) structures. As the “glue” in the CNS, ECM plays a pivotal role in synaptic plasticity, biophysical protection, and cell signaling [59, 60]. PNNs are mainly composed of hyaluronic acid, and chondroitin sulfate proteoglycan (CSPG), and condense in a reticular fashion around the fast-spiking parvalbumin (PV)-expressing GABAergic interneurons primarily [61]. PNNs form and mature at different times in distinct brain regions during development [62], with the earliest cortical maturation in adult rodents. Interestingly, microglia appear to strip PNN directly from the surface of the neuron or indirectly by removing fragments of PNN cleaved by matrix metalloproteinases (MMP) [63, 64], whereas inhibition of microglia can effectively restore PNN [65]. This evidence suggests that microglia are an important element in the dynamic homeostasis of the PNN in healthy states as well as in the remodeling of the PNN in disease states. Nguyen *et al.* further found that IL-33 is a key factor in microglia-mediated ECM remodeling [66]. Neurons in the hippocampus recruited microglia through high expression of IL-33, which then phagocytosed the ECM around the synapses, thereby enhancing the plasticity of dendritic spines and the synapses' capacity to develop memory traces. Subsequently, the researchers analyzed microglia at the transcriptional level and identified the upregulation of a variety of genes involved in phagocytosis after IL-33 treatment [66]. Moreover, microglia-mediated degradation of PNN also plays an important role in some pathological conditions. For example, PNNs around synapses are degraded after peripheral nerve injury in a microglia-dependent manner, reducing inhibitory synaptic input and enhancing the activity of projection neurons, thus promoting pain behavior [67]. In addition, reductions in perineuronal nets (PNN) have been observed in several CNS disorders, including Alzheimer's disease (AD) [68], Huntington's disease [65], stroke [69], multiple sclerosis (MS) [70], traumatic brain injury (TBI) [71], which are usually involved in the activation of microglia Altogether, current evidence indicates that microglia plays a regulatory role in the modification of PNN and indirectly affects synaptic plasticity. When the balance of microglia regulation of PNN is abnormal, it will eventually lead to the occurrence of pathological changes in the CNS.

## 3 SYNAPSE

Synapses are specialized cell-to-cell connections that enable fast, point-to-point information transfer between neurons [72]. This is achieved through the transmission of electrical signals (electrical synapses) and chemical transmitters (chemical synapses) between neurons [73].

### 3.1 Synapse Structures

Classical synaptic structures include presynaptic specialization, postsynaptic specialization, and synaptic cleft separating them. Presynaptic specializations can only be neuronal (mostly axons) and may be assembled by dendrites in specific areas of the brain, such as the thalamus [74]. Postsynaptic specializations in the brain are also predominantly neuronal, but some glial cells can also receive presynaptic signals [75]. Outside of the brain, virtually any cell type can elaborate postsynaptic specializations. A single axon is lined with numerous presynaptic specializations, each of which can form either single synapses with the postsynaptic specializations or multiple synapses with multiple postsynaptic specializations [76]. This ensures the efficiency and accuracy of CNS information transmission.

### 3.2 Synaptogenesis and Synapse Elimination

Synaptogenesis and synaptic elimination are under the control of a series of elaborate programs [72]. In humans, it is currently accepted that exuberant synaptogenesis occurs from late pregnancy to 2-year postnatal, followed by a period of net synapse elimination, during which the number and density of synapses decrease to half their maximum value [77]. A phase of net synapse elimination occurs late in childhood and continues into early or middle adolescence [78]. Synapse number stabilizes in adulthood [79] and declines slightly in the elderly brain [77].

#### 3.2.1 Synaptogenesis

Synaptogenesis occurs throughout the life cycle. Recent studies show that neurons use a group of proteins to mediate the initiation of synapse formation and achieve precise presynaptic and postsynaptic alignment, including synaptic cell adhesion molecules (SynCAM); the neurexin–neuroligin pairs, cadherins, and other structurally unrelated proteins such as Teneurins [80]. In addition, synapse formation is regulated by presynaptic neurotransmitters, mechanical factors, astrocytes, ECM, and microglia (the role of microglia is discussed in detail in 4.1). There is still controversy about whether presynaptic neurotransmitters contribute to the synapse formation. Studies have shown that even an individual neuron can form distinct synapses with corresponding postsynaptic receptors due to the release of different types of neurotransmitters [81, 82]. This suggests that presynaptic neurotransmitters instruct postsynaptic specialization, although the underlying mechanisms are unclear. Consistent with this hypothesis, rapid local release of caged glutamate or GABA using photolysis induces functional synapses [83]. However, other studies have shown that abolishing neurotransmitter release using genetic tools does not prevent the formation of synapses [84, 85]. Therefore, whether and how presynaptic neurotransmitters regulate synapse formation is still a question that remains unsolved. The formation and stability of dendritic spines are essential for synapse formation. Bin/Amphiphilin/Rvs (BAR) domain proteins mediate dendritic membrane deformation, which initiates spine formation. Cytoskeletal proteins, including regulatory actin filaments (F-actins), support the structural stability of dendritic spines [86].

The function of synapses is strongly influenced by the microenvironment, including astrocytes, the ECM, and microglia. Therefore, many studies incorporate astrocytes and the ECM into the structural paradigm of synapses, known as “triple synapses” and “tetragonal synapses”, respectively [87, 88]. Astrocytes participate in synaptic development and formation through elaborate perisynaptic processes. Astrocytes cover synapses in a tiled manner and regulate synapse formation through direct contact with neurons [89]. Furthermore, signaling molecules secreted by astrocytes such as cholesterol, Hevin/SPARC, heparan sulfate proteoglycans Glypican 4 and 6 (Gpc4 and 6), TNF-α, and thrombospondins promote synaptic development and maturation [90]. The main components of the ECM, including CSPG, tenascin R (TNR) glycoprotein, and hyaluronic acid [91], interweave into structural scaffolds that maintain synaptic stability. The ECM also provides nutrient molecules for neurons and enables cell-to-cell communication with neurons and glial cells by binding membrane-bound molecules [92].

#### 3.2.2 Synapse Pruning

Pruning of overproduced or unnecessary synapses is essential to promote the renewal and maturation of synapses and the improvement of CNS function [93]. In contrast, synapse over-pruning or defective pruning causes the pathogenesis of numerous neurological diseases, *e.g*., AD, MS, epilepsy, and schizophrenia [94].

Synapse pruning peaks during postnatal development and is generally considered to be activity-dependent. The most widely studied examples are climbing fibers at the cerebellar Purkinje cell synapses, motor input at the neuromuscular junction, and retinal ganglion cell input at the retinal dorsolateral geniculate nucleus [95, 96]. Synapses also turn over in the mature brain, although it is much less active than in postnatal development. Live imaging shows that 40% of dendritic spines are replaced every 5 days in sensory and motor cortex neurons in mice, while ∼60% of dendritic spines are stable [97]. Synapse clearance in adulthood is thought to be experience-dependent. For example, in the hippocampus, nearly all spines turn over every two weeks in adults [98], and sensory experience increases synaptic turnover with unknown mechanisms. Importantly, newly formed dendritic spines exhibit more enduring stability during learning processes, which favors memory storage in the brain [99].

At the cellular and molecular levels, synapse pruning is regulated by a variety of immune molecules (including pentapeptide and MHC I), astrocytes, and microglia. Mice lacking Neuronal pentapeptide 1(NP1), NP2, and NP receptors have defects in early synapse refinement in the retina and dorsal geniculate nucleus [100]. MHC I was the first immune-related molecule found to be involved in synapse elimination during development. MHC I expressed by neurons is enriched in brain regions dependent on active synapse remodeling [101]. Downregulation of MHC I expression by the developing neuromuscular junction affects postnatal synapse elimination [102]. Astrocytes express many phagocytic proteins to mediate synapse pruning. For instance, the upregulation of phagocytic receptors MEGF10 and MERTK is involved in removing unnecessary synapses in the developing retinol-producing system and the adult hippocampus [103, 104]. In addition, SPARC expressed by astrocytes triggers a cell-autonomous synapse elimination program in cholinergic neurons, which promotes substantial clearance of neuromuscular junctions and axonal branches [105]. The role of microglia in synapse pruning is elaborated in 4.1.

### 3.3 Synaptic Plasticity

Generalized synaptic plasticity includes changes in the number of synapses and the efficiency of transmitter transmission at existing synapses; mechanisms involving changes in synapse numbers were discussed in detail above. Donald Hebb initially proposed the concept of changes in synaptic connection strength and the famous long-term potentiation (LTP) phenomenon in 1949 [106]. Since then, more forms of synaptic plasticity have been gradually discovered, including short-term potentiation, long-term depression, and homeostatic scaling (cell-autonomous and global) [107]. In general, synaptic plasticity is determined by presynaptic plasticity and postsynaptic plasticity, although in various forms [108].

Presynaptic plasticity usually refers to the activity-dependent regulation of neurotransmitter release. This process is regulated by Voltage-gated Ca^2+^ channels (Cav), axon-synthesized proteins, CAMs, and presynaptic structural changes. Calcium influx caused by the opening of calcium channels triggers the fusion of synaptic vesicles near Cav with active zone (AZ) membranes. It promotes the release of neurotransmitters when action potentials are conducted to presynaptic membrane terminals [109]. Axons can synthesize a variety of proteins, including cytoskeletal proteins (such as neurofilament subunits and β-actin) and proteins associated with synaptic vesicle cycling, such as MUNC18-1, which are essential for neurotransmitter transmission [110]. In addition, CAMs play an important role in neurotransmitter transmission. For example, the loss of neurofilament proteins impairs synaptic transmission by reducing presynaptic calcium influx [111]. Several other studies demonstrated that the presynaptic terminal undergoes a series of structural changes, such as the insertion or removal of AZ sites and redistribution of vesicle pools, which all affect the efficiency of neurotransmitter transmission [112].

Postsynaptic plasticity is also influenced by many factors, including the number and nature of receptors and the structural changes of postsynaptic terminals. Postsynaptic receptors continuously undergo diffusion and recombination after capture. For example, rapid surface diffusion and redistribution of α-amino-3-hydroxyl-5-methyl-4-isoxazole-propionate (AMPA)-type glutamate receptors (AMPARs) are involved in regulating frequency-dependent short-term plasticity and LTP, respectively [113, 114]. Dendritic spines change in number, shape, and size during development and synaptic activity, which is regulated by cytoskeletal proteins such as F-actin, CAMs such as integrins, cadherins, and ECM [115]. In conclusion, synaptic plasticity is a delicate process involving multiple links. A small error can lead to impaired plasticity, followed by neurodegenerative diseases (*e.g*., AD, Parkinson's disease (PD), and Huntington’s disease), neurodevelopmental disorders (*e.g*., autism and schizophrenia), and receptor-related diseases such as NMDAR (N-methyl-D-aspartate receptor) encephalitis [80, 90, 116].

## 4 ROLES OF MICROGLIA ON SYNAPSES IN PHYSIOLOGICAL CONDITIONS

### 4.1 Roles of Microglia on Synapses in the Developing CNS: Synaptogenesis and Synapse Pruning

Microglia promote the formation and maturation of synaptic connections during CNS development and also find and prune redundant or “weak” synapses. The dual roles ensure the correct connection and functional maintenance of synapses during development.

#### 4.1.1 Microglia can Promote Synaptogenesis and Maturation

##### 4.1.1.1 Microglia Promotes Synaptogenesis and Early Network Wiring

As mentioned above, microglia provide trophic support for synapse formation during brain development. The conditioned culture medium of primary microglia isolated from embryonic rat brain promote axonal growth *in vitro*. This was inhibited when antibodies to ECM protein thrombospondin were added. These results suggest that microglia regulate axonal growth through the release of thrombospondin [117]. Another *in vitro* study demonstrated that the conditioned medium of microglia culture promotes the proliferation of cerebellar granule cells through mitogen-activated protein kinase-, phosphatidylinositol-3-kinase/Akt-s, and δ-Notch signaling pathways [118].

Microglia are densely distributed in the subventricular zone from day 1-10 postnatal, which is one of the main regions that stimulates neurogenesis by releasing IL-1β and IL-6 [119, 120]. *In vivo*, multiphoton imaging revealed frequent contact between microglia and dendrites of approximately 2/3 pyramidal neurons in the developing somatosensory cortex, which directly induces filopodia formation during P8 to P10 [121]. Interestingly, it seems that only one or a few specific subsets of microglia promote synaptogenesis. CD11c^+^ microglia are a typical subset that promotes the axonal growth of corticospinal motor neurons by secreting IGF1 in the neonatal brain [122].

##### 4.1.1.2 Microglia Promote Synapse Maturation

Neurons express the chemokine CX3CL1 (fractalkine), which acts as a “Find Me” signal to attract microglia by binding to CX3CR1 on the surface of microglia, which affects synaptic maturation [123]. Loss of CX3CR1 leads to impaired synaptic maturation and functional defects. CX3CR1 knockout mice (Cx3CR1-KO) have an increased number of immature synapses in the hippocampus and show a decline in cognitive function [124]. They also show impaired synaptic maturation in the thalamic barrel cortex. This is due to the delayed recruitment of microglia to mature sites of thalamocortical synapses [125]. In addition, specific mediators released by microglia are involved in regulating synaptic maturation, including BDNF and TNF-α. Specific knockdown of BDNF in microglia of CX3CR1 (CreER) mice demonstrated that BDNF is essential for synapse formation and expression of synaptic proteins (including AMPAR and NMDAR subunits) [126]. Meanwhile, TNF-α can regulate the transmission of synaptic transmitters by promoting the expression of AMPAR and NMDAR and the endocytosis of GABAA R, thereby promoting the maturation of synapses [127, 128].

#### 4.1.2 Microglia Refine Neural Circuits *via* Synapse Pruning

Microglia monitor and phagocytose synapses through frequent contact with synapses for the refinement of neural circuits [120]. Physical contact between microglia and synapses was first detected in mice using two-photon time-lapse imaging; microglia make transient, repetitive contact with synapses in layers II-III of the somatosensory and visual cortex [129]. Multiple subsequent studies further revealed direct membrane-membrane contacts between microglia and various parts of the synapse (dendrites, spines, or presynaptic elements), with the elimination of some synapses that are directly in contact with microglia [121, 124, 130].

Molecular signals are involved in mediating selective phagocytosis of synapses by microglia. Some synapses express “Find me” and “Eat me” signals to induce phagocytosis and clearance of microglia. Meanwhile, some synapses protect themselves from being devoured by microglia by expressing “Don't Eat Me” signals.

##### 4.1.2.1 “Find me” Signals

###### 4.1.2.1.1 ATP/ADP-P2Y12 Signaling

The P2Y12 receptors are selectively expressed in nonpathological microglia during development and induce the recruitment of microglia by responding to adenosine diphosphate (ADP), a hydrolysate of adenosine triphosphate (ATP) [131]. For example, local neurons release ATP during ocular dominance plasticity, which quickly degrades to ADP and activates P2Y12 receptors, thereby inducing its phagocytosis by microglia [132]. P2Y12 receptors have also been shown to be involved in the phagocytosis of spinal cord axons by microglia [133].

###### 4.1.2.1.2 CX3CL1-CX3CR1 Signaling

CX3CL1/CX3CR1 signaling is also involved in synaptic pruning during development. CX3CL1 is expressed on cortical neurons, and the metalloproteinase ADAM10 cleaves CX3CL1 to a secretory form, which attracts microglia and promotes synaptic phagocytosis [134]. CX3CR1 knockout mice have reduced synaptic phagocytosis by microglia [135], and the mice develop autism-like behavior [136].

##### 4.1.2.2 “Eat me” Signals

###### 4.1.2.2.1 Complement

Apoptotic, immature, or weaker synapses express the cascade initiation protein C1q, which subsequently leads to the deposition of the downstream protein, C3 [137]. Activated C3 (iC3b/C3b) acts as an “Eat Me” signal to activate CR3 on the surface of microglia and further mediate the phagocytosis of target synapses by microglia. Loss of CR3, C1q, or C3 reduces synaptic pruning, which leads to dissociation impairment of geniculate projection detachment in the developing retina [138].

###### 4.1.2.2.2 Phosphatidyl Serine

Phosphatidylserine (PS) is another “Eat Me” signal which is generally located in the inner lobe of the plasma membrane. It can be exposed to the cell surface, including reversible exposure on the surface of living cells due to calcium-activated phosphatidylserine scramblases [139], and irreversible exposure to apoptotic cell surfaces [140]. Phosphatidylserine is recognized by microglia and mediates neuronal phagocytosis. Microglia can either directly recognize PS by expressing TREM2 or G protein-coupled receptor 56 (GPR56) [141, 142], or indirectly bind to PS through opsonins GAS6 or Milk Fat Globule-EGF Factor 8 protein (MFG-E8) [143, 144].

###### 4.1.2.2.3 Calreticulin

Cell surface calreticulin is an alternative “eat me” signal that interacts with low-density lipoprotein receptor-associated protein 1 (LRP1) on microglia to initiate the phagocytic process [145]. Calreticulin is mainly located in the endoplasmic reticulum under physiological conditions and is released to the cell surface during stress and apoptosis in this organelle [146]. Blocking calreticulin on the surface of neurons or LRP1 of microglia inhibits lipopolysaccharide-induced phagocytosis of microglia to live synapses [147].

##### 4.1.2.3 “Don’t Eat Me” Signals

###### 4.1.2.3.1 CD47

CD47 is a transmembrane protein expressed on the surface of neurons and avoids excessive phagocytosis by recognizing signal regulatory protein α (SIRPα) on the surface of microglia. For example, CD47 preferentially localizes to the more active synapses in the dorsolateral geniculate nucleus of the thalamus at the peak of synaptic pruning during mouse development and prevents them from being engulfed by microglia [148]. CD47-deficient mice show increased microglial phagocytosis of retinal ganglion cells and a sustained decrease in the synaptic number [138]. CD47 expression on myelin debris avoids clearance by microglia *via* SIRPα and contributes to Wallerian degeneration [149].

###### 4.1.2.3.2 Sialic Acid

The surface of healthy neurons has a high density of sialic acid residues on their glycoproteins and glycolipids. These sialic acid residues inhibit the phagocytosis of microglia by binding to sialic acid-binding immunoglobulin-type lectin (Siglec) receptors on microglia [146]. For instance, Siglec11 interacts with sialic acid residues of neurons to inhibit neuronal phagocytosis *in vivo* [150]. Sialic acid residues can also prevent the erroneous phagocytosis of healthy neurons by inhibiting the binding of opsonins C1q, C3b, and Galectin-3 [151]. Sialidase secreted by activated microglia leads to the inactivation of co-cultured PC12 cells due to the binding of de-sialylated PC12 cells to Galectin-3, leading to its phagocytosis by microglia *in vitro* [152].

#### 4.1.3 Synapses promote Microglial Development and Motility

**Fig. (1):**
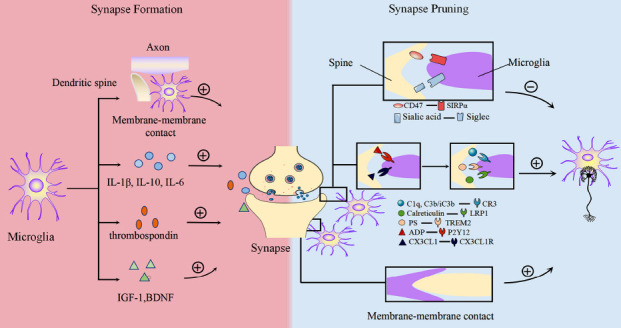
Microglia-synapse interactions in the developing CNS: synapse formation and pruning. Microglia promote neurogenesis and synapse formation through membrane-membrane contact and the release of cytokines during development. Frequent contact between microglia and dendrites directly induces filopodia formation. Microglia that accumulate in SAZ release IL-1β and IL-6 to promote neurogenesis. *In vivo* and *in vitro* experiments demonstrate that microglia promote synapse formation by producing IL-10, IGF1, and BDNF. Microglia induce synaptic pruning primarily through the expression of specialized receptors. Redundant or weak synapses express “Find me” and “Eat Me” signals such as C3b, iC3b, PS, ADP, and CX3CL1, which induce microglial phagocytosis by activating corresponding receptors on microglia. Direct membrane-membrane contacts between microglia and synapses (dendrites, spines, or presynaptic elements) also facilitate synaptic clearance. However, some synapses express “don't eat me” signals such as CD47 and sialic acid to avoid being engulfed by microglia.

As mentioned above, microglia remain nonreactive in physiological conditions. A variety of neurotransmitters released from synapses affect microglia movement. Glutamate can be transmitted to increase microglia motility directly or by increasing the release of ATP from neurons. The inhibitory neurotransmitter gamma-aminobutyric acid (GABA) reduces microglia motility and overall velocity *in vitro* [153]. Moreover, time-lapse two-photon imaging in acute brain slices confirms the effect of LTP on microglia motility. Induction of LTP in the hippocampus increases microglia motility and their contact with dendritic spines [154] (Fig. **1**).

### 4.2 Roles of Microglia on Synapses in the Mature CNS: Synaptic Plasticity

Synaptic plasticity is the foundation of learning and memory, which are inseparable from the regulation of microglia. Microglial depletion induced by PLX 5622 (a selective inhibitor of CSF1R) results in impaired synapse maturation in the hippocampus, decreased density, and learning and memory deficits in mice, and the effects were restored by microglial supplementation [155]. Below are the molecular mechanisms through which microglia regulate synaptic plasticity.

#### 4.2.1 BDNF

Microglia promote learning-dependent synapse remodeling by releasing BDNF [126], which regulates synaptic transmission and plasticity in many brain regions by binding to its receptor tropomyosin-associated kinase B (TrkB) [156]. For example, BDNF induces LTP in the hippocampus and insular cortex [157, 158]. Increased BDNF/TrkB signaling in the dorsal horn of the spinal cord induces LTP and participates in neuropathic pain by activating peripheral microglia [159].

#### 4.2.2 ATP

Two-photon imaging shows that contact of inactive microglia with dendrites increases synapse activity and enhances the synchrony of neuronal population activity *in vivo* [160]. Adenosine triphosphate released during high neuronal activity enhances microglia motility, which enhances dynamic synapse monitoring [153]. Furthermore, ATP promotes the release of microvesicles (MVs) from microglia by activating P2X7 receptors on the surface of microglia. Neurons exposed to MVs spontaneously release glutamate, which triggers an increase in the frequency of miniature excitatory postsynaptic currents (mEPSCs) [161]. The release of ATP from microglia activates P2Y1 receptors on the surface of astrocytes, which affects glutamate receptor release and increases EPSCs [162].

#### 4.2.3 Glutamate Receptors

Microglia also alter synaptic activity by affecting glutamate receptor expression. The addition of conditioned medium from microglia to cortical slice cultures results in a greater amplitude and longer duration of NMDAR-mediated EPSC frequency *in vitro* [163]. Meanwhile, the addition of microglia to neuronal cultures decreases the expression of the AMPA receptor (GluA1) [164].

#### 4.2.4 Cytokines and Proteases

**Fig. (2):**
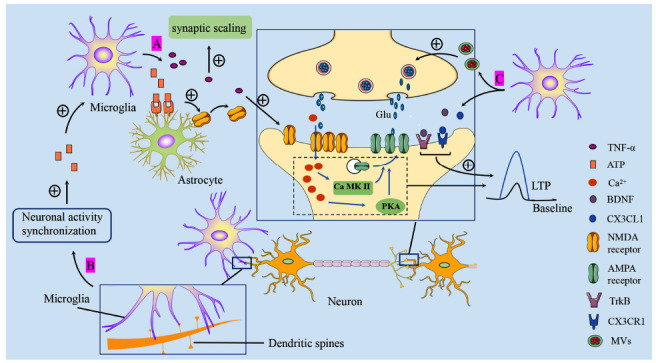
Microglia-synapse interactions in AD: The state and transcriptional properties of microglia are altered by the induction of abnormally deposited oligomeric Aβ. Microglia can effectively phagocytose Aβ oligomers with the assistance of TREM when new Aβ oligomers are generated while performing their anti-inflammatory effects. Microglia form a “barrier” around Aβ plaques to prevent Aβ from damaging surrounding cells (including synapses) and limit the growth of fibers within Aβ plaques. Meanwhile, microglia can secrete hydrolases that promote the degradation of Aβ plaques. Synapses damaged by Aβ can be recognized and phagocytosed by microglia through the complement pathway, thereby reducing the spread of damage along axons and limiting the damage near plaques. With the accumulation of tau and NFT, the pro-inflammatory effects of microglia gradually enhanced, the phagocytosis of Aβ by microglia gradually weakened; microglia-induced peripheral astrocytes adopting a neurotoxic A1 phenotype; The reduction or deficiency of SIRPα increases the abnormal pruning of synaptic structures by microglia, further leading to the loss of neurons and synapses.

Microglia express cytokines and proteases that are involved in the regulation of synaptic plasticity. TNF-α enhances synaptic efficacy by increasing the surface expression of glutamate receptors [165] and acts as an effector to regulate synapse scaling [166]. CX3CL1-CX3CR1 plays a role in inhibiting excitatory currents and inducing LTP *in vitro* [167, 168]. LTP is severely impaired in CX3CR1-deficient mice, and cognitive performance is negatively affected [168]. Besides, proteases produced by microglia, such as MMP24 and ADAM7, reduce the levels of PSD-95 and AMPA receptor GluA2, thereby reducing the frequency of mEPSC [169] (Fig. **2**).

## 5 ROLES OF MICROGLIA ON SYNAPSES IN CNS DISORDERS

### 5.1 Roles of Microglia on Synapses in Pathological Conditions

Given the role of synaptic dysfunction in numerous neurological disorders and the critical role of microglia in the regulation of synaptic structure and function in physiological conditions, it is likely that alterations in microglia function may contribute to some of the pathology of neurological disorders. Indeed, microglia dysfunction has in recent years been shown to be a common crossroads in many neurological disorders such as neurodegenerative diseases (*e.g*., AD, PD, and MS); stroke, traumatic brain injury, and epilepsy [170-175]. Misfolded proteins and neuronal damage result in the activation of microglia *via* damage-associated molecular patterns (DAMPs), inducing persistent inflammation, oxidative stress, and phagocytosis, which are closely associated and mutually reinforcing, leading to synaptic damage and neuronal loss [176-178]. Reactive microglia release inflammatory mediators and free radicals to induce synapse and neuron damage directly. Meanwhile, microglia-mediated inflammation and oxidative stress can promote their recognition of inflammation-associated phagocytic receptors, such as “find me” and “eat me” signals to exert pathological phagocytosis of synapses.

#### 5.1.1 Inflammation

In general, Inflammatory cytokines (*e.g*., IL-1β, TNF-α, and IL-6) have little neurotoxicity. Still, they can mediate neurotoxicity indirectly by stimulating microglia to release glutamate in an autocrine/paracrine manner, resulting in the formation of neurogenic beads, *i.e*., focal bead-like swelling in dendrites and axons [179]. Moreover, TNF-α has been shown to promote glutamate release through opening connexin 32 hemichannels (Cx32 HCs) on the microglia [180]. Cxs and pannexins (Panxs) are two proteins that form gap junctions and pannexons between microglia and other brain cells, respectively, and are responsible for part of the communication between microglia and other brain cells and for the spread of microglia-mediated inflammation under pathological conditions [181]. For instance, IL-1β activates Cx43 HCs in astrocytes or Panx1 channels in microglia, inducing the release of ATP [182], which leads to the activation of P2X7R and P2X4R, which can further activate NRLP3 pro-inflammatory inflammasome pathway in microglia to exacerbate inflammation responses [177].

#### 5.1.2 Oxidative Stress

Reactive microglia can also directly kill neurons by activating NADPH oxidase 2 (NOX2) and expressing iNOS (inducible nitric oxide synthase), with the production of ROS and NO [183, 184]. NOX2 and ROS also act as drivers of excessive inflammation in response to nerve injury and may impede the reduction of inflammation *in vivo*. For example, ROS triggers nuclear factor kappa B(NF-κB), interferon regulatory factor 1, STAT3, and p53, triggering the release of pro-inflammatory mediators [185]. To resist the damaging effects of inflammation and oxidative stress, the organism has developed several defense mechanisms. In particular, Nuclear factor E2-related factor 2 (Nrf2) is a key modulator of two important cytoprotective pathways, anti-inflammatory and antioxidant. It influences the anti-inflammatory changes in a variety of brain injuries such as TBI, subarachnoid hemorrhage, and neurodegenerative diseases [186-188]. Nrf2 upregulates anti-oxidant and anti-inflammatory downstream genes *via* interaction with an antioxidant response element (ARE), thereby restricting the expression of pro-inflammatory markers in microglia for neuroprotection [184].

#### 5.1.3 Phagocytosis

As phagocytes, reactive microglia upregulate phagocytosis-associated receptors (*e.g*., CR3, MerTK, Axl, and TREM2) and mediate pathologic phagocytosis in CNS disorders [176], including reduced phagocytosis and excessive phagocytosis. Insufficient phagocytosis of harmful substances and erroneous phagocytosis of living cells by microglia exacerbate the progression of the disease (*e.g*., in the penumbra area of ischemic stroke [189] or in neurodegenerative diseases [58]). Nevertheless, microglia also act as protectors in neurological disorders, as shown by anti-inflammatory effects, removal of lesional tissue, and trophic support for neurological repair [11, 190, 191]. Notably, the role of microglia in different diseases is far more complex than currently known due to their high heterogeneity and differences in the pathogenesis of various diseases. Furthermore, it is not yet fully understood how microglia affect synaptic function. Next, we will discuss the known connections between microglia and synapses in these diseases (Table **1**), and we aim to highlight new potential targets for therapeutic consideration by elucidating the effects of microglia on synaptic function in disease.

### 5.2 Neurodegenerative Diseases

#### 5.2.1 AD

**Fig. (3):**
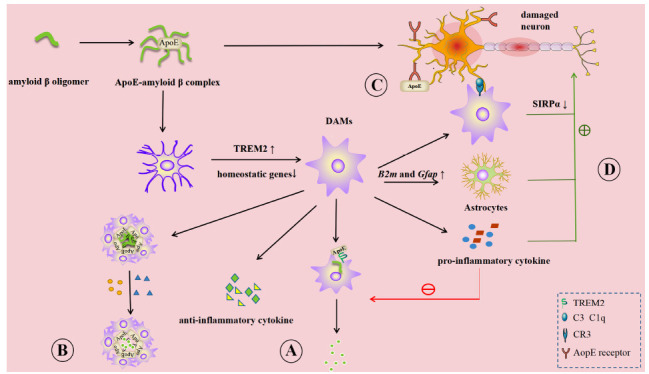
Microglia-synapse interactions in AD: (**A**) The state and transcriptional properties of microglia are altered by the induction of abnormally deposited oligomeric Aβ. Then, microglia can effectively phagocytose Aβ oligomers with the assistance of TREM when new Aβ oligomers are generated while performing their anti-inflammatory effects. (**B**) Microglia form a “barrier” around Aβ plaques to prevent Aβ from damaging surrounding cells (including synapses) and limit the growth of fibers within Aβ plaques. Meanwhile, microglia can secrete hydrolases that promote the degradation of Aβ plaques. (**C**) Synapses damaged by Aβ can be recognized and phagocytosed by microglia through the complement pathway, thereby reducing the spread of damage along axons and limiting the damage near plaques. (**D**) With the accumulation of tau and NFT, the pro-inflammatory effects of microglia gradually enhanced, the phagocytosis of Aβ by microglia gradually weakened; microglia-induced peripheral astrocytes adopting a neurotoxic A1 phenotype; The reduction or deficiency of SIRPα increases the abnormal pruning of synaptic structures by microglia, further lead to the loss of neurons and synapses.

AD is one of the most common neurodegenerative diseases and is characterized by a progressive decline in cognitive function, memory, and speech ability. It can be accompanied by hallucinations, delusions, and seizures in severe cases [192, 193]. The pathological features of AD are the deposition of extracellular amyloid beta peptide (Aβ) and intracellular neurofibrillary tangles (NFT), which interact directly with synaptic proteins (such as NMDAR) and cause excessive activation, which leads to high levels of glutamate and a subsequent cascade of oxidative stress responses [194, 195]. It was long believed that oxidative stress and inflammation are the pathogenesis of AD, and synaptic changes are the core of the development of AD. This change is related to the modification of cognitive function in AD patients [196, 197]. Microglia dysfunction is considered to be the main culprit of neuronal damage in AD (Fig. **3**) and may induce the failure of synaptic pruning, which is closely related to Aβ plaques [198].

Abnormally deposited oligomeric Aβ induced microglia response and aggregated microglia to Aβ plaques [199]. The states and transcriptional identity of microglia have alerted during this progress. In mice models of AD, it is found that microglia differentiated from ” homeostasis ” to DAMs [170, 200]. This progress has two sequential phases, the first involving decreased expression of homeostatic genes (*e.g*., P2RY12/P2RY13, CX3CR1), and the second involving activation of metabolic and phagocytic pathways, which depends on TREM2 [170]. The increased phagocytic activity of DAMs is believed to help phagocyte and clear pathological proteins more effectively in the area, which may play a protective role in the AD brain. DAM also has pro-inflammatory and anti-inflammatory properties, and increased pro-inflammatory proteins of DAM are positively correlated with the accumulation of tau and NTF [49]. In addition, Daniel *et al*. demonstrated that microglia progressively differentiate from a homeostatic state into four different states (DAMs, interferon-responsive microglia, cycling microglia, MHC-II expressing microglia) after using TREM2 agonists in mice [201]. MGnD, which is associated with a variety of degenerative diseases, has also been identified in AD mouse models and AD humans, and it is thought to partly overlap with the role of M1 in regulating the development of AD [50]. In addition, HAM was characterized in frozen human frontal cortex samples, and in AD whole-tissue RNA profiles, HAM genes overlapped with natural aging genes and contained genes related to phagocytosis [202]. Overall, although many different microglial states have been identified in samples and models of AD, their effects are inseparable from phagocytic, pro-inflammatory, and anti-inflammatory.

When new plaques are generated, TREM2 enables microglia to rapidly anchor and tightly wrap around the plaque. This is due to the strong affinity of TREM2 with Apolipoprotein E (ApoE) in Aβ plaques; furthermore, ApoE interacts with Aβ and promotes Aβ aggregation to help microglia localize to Aβ plaques [190, 203, 204]. Microglia form a “barrier” around Aβ plaques, which blocks Aβ damage to surrounding cells, including synapses, and limits the growth of fibers within Aβ plaques [190, 205]. At the same time, microglia can secrete hydrolases that promote the degradation of Aβ plaques [206]. The phagocytosis of microglia also plays a role in this process. Microglia can express receptors that promote Aβ phagocytosis, such as class A scavenger receptor and CD36. In addition, several microglial states, including but not limited to DAM and HAM, also have phagocytosis. However, the role of microglia in plaque phagocytosis is still controversial. Microglia do not affect the size of the original plaque *in vivo* and *in vitro* [207]. However, some researchers believe that microglia can phagocytose nascent Aβ oligomers [190, 208], thereby reducing the formation of new plaques.

Frances A. Edwards' group proposes that microglia remove damaged synapses by phagocytosis during AD [203]. The response of microglia to Aβ plaques and tau may reduce the spread of damage along axons and limit damage near plaques. This idea is supported by the discovery of synaptic proteins within microglia and synaptic components within lysosomes in microglia [203, 205, 209]. However, the protective effect of microglia gradually diminishes as the disease progresses. With the accumulation of tau and NFT, the pro-inflammatory effect of microglia is gradually enhanced [49]. Phagocytosis of microglia is gradually weakened under the continuous stimulation of pro-inflammatory factors, including IL-1β. Furthermore, the clearance of Aβ is not enough to counteract the formation of new plaques, and abnormal phagocytosis leads to the continued loss of synapses and neurons [198, 199]. After selective inhibition of some complements (including C3, C1q, CR3) in mice with frontotemporal dementia caused by mutant tau, researchers found that synaptic and neuronal loss was reduced compared with mice without inhibition of complement. This suggests that tau-induced abnormal synapse pruning and neuronal loss in AD are closely related to complement-mediated microglial phagocytosis [176]. Although the decrease in synaptic density is an important pathogenesis of AD, direct evidence of microglia-mediated synaptic pruning failure in neurodegenerative diseases has been rarely reported. Some articles have provided indirect evidence. For example, it is found that the elevation of “Eat me” signals (C3, C1q) in the hippocampus synapses, the vulnerable area of AD, may mediate the excessive phagocytosis of synapses by microglia and lead to the failure of synaptic pruning and the loss of synapses [207]. Until recently, Ding *et al.* found that Microglial SIRPα is downregulated in AD pathology [210]. SIRPα is a transmembrane protein that binds to its ligand CD47 [211]. The SIRPα-CD47 axis has been shown to mediate negative regulation of synaptic pruning in the CNS [212]. The reduction or deficiency of Sirpα increases the phagocytosis of microglia to synaptic structures, which ultimately leads to the aggravation of cognitive disorder in AD patients. Plaque hydrolase expression is reduced in microglia, which leads to a decreased ability to clear Aβ oligomers and damaged synapses and aggravates the accumulation of Aβ plaques. At the same time, microglia continuously produce pro-inflammatory cytokines, glutamate, cathepsin B, superoxide, or nitric oxide (NO), which aggravate the damage of synapses [178, 206, 213]. And Aβ can directly activate Cx43 HCs in microglia and astrocytes, promoting the release of ATP and glutamate and leading to neuronal death [214]. Furthermore, it was observed that the level of Nrf2 is reduced in AD patients [215]. The lack of Nrf2 was shown to increase the number of reactive microglia around Aβ and promote their transition to a pro-inflammatory phenotype, which further exacerbates the inflammation [188]. It is worth mentioning that microglia with high expression of DAM-signature genes (*i.e*., *G1qa* and *C1qb*) induce peripheral astrocytes to adopt neurotoxic A1 phenotype by increasing A1 astrocytes signature gene expression (*B2m* and *Gfap*) [216], A1 astrocytes are highly neurotoxic, which will further lead to the loss of neurons and synapses [217].

#### 5.2.2 PD

PD is a neurodegenerative disorder characterized by motor deficiencies such as rigidity, bradykinesia, and resting tremor [218]. Neuropathologically, PD is characterized by progressive degeneration of dopaminergic (DA) neurons in the substantia nigra pars compacta and abnormal accumulation of intraneuronal α-synuclein (α-syn), termed Lewy bodies (LBs) and Lewy neurites [219].

α-syn is a small (14 kDa) cytosolic protein. In physiological conditions, α-syn monomer is involved in neurotransmitter release by regulating the maturation as well as the release of synaptic vesicles [220]. Under pathological conditions, α-syn misfolds, and aggregates into multiple soluble oligomeric species and eventually insoluble amorphous or fibrillar amyloid-like assemble [221]. The accumulation of a-syn in presynaptic terminals is the initial event of PD, and the accumulated a-syn impedes SNARE-mediated vesicle fusion and neurotransmitter release [9]. In addition, the high energy demand of striatal dopamine neurons makes mitochondrial activity vulnerable to stressors such as a-syn accumulation, leading to mitochondrial dysfunction and energy depletion in dopamine neurons [222]. As the primary compartment of a-syn pathology, synapses are involved in the spread of a-syn between cells and brain regions [223]. In this process, extracellular a-syn affects microglia to turn them into reactive microglia, which has been supported by brain autopsy results in PD patients and PD animal models [224, 225]. It is suggested that the possible mechanisms are that a-syn promotes the reactive state of microglia and activates downstream pro-inflammatory signaling cascades by activating STAT3, TLR2, TLR4, NLRP3 inflammasome axis and inducing mitochondrial dysfunction [226-228]. A study profiled single-nuclei transcriptomes of post-mortem midbrain from six idiopathic PD patients and identified PD-associated microglia revealing a pro-inflammatory trajectory characterized by elevated levels of IL-1β, GPNMB, and HSP90AA1, have been demonstrated to be involved in inflammation and promote neurodegeneration [54]. Besides, reactive microglia were demonstrated to promote ROS production through activation of NOX2, thereby exacerbating neurotoxicity in α-syn-treated midbrain cultures [229]. At the same time, Nrf2 activity was significantly reduced in the MPTP model [230]. In Nrf2-knockout mice with MPTP treatment, basal ganglia show a more severe dopaminergic dysfunction than wild type. And microglia showed more intense proliferation and higher levels of pro-inflammatory markers (*e.g*., iNOS, IL-6, and TNF-a) [231].

Although the inflammatory state of microglia in PD patients has been revealed by the application of single-nuclei transcriptomes, the function of reactive microglia in the pathogenesis of PD is still unconfirmed. On the one hand, microglia can inhibit the spread of a-syn by phagocytosing a-syn and damaging synapses through TLR4 and autophagic pathways [232, 233]. What's more, a study demonstrated that a-syn monomer induces the anti-inflammatory state of microglia by decreasing ERK/NF-κB activation and increasing PPARγ expression, thereby promoting extracellular a-syn removal [234]. Co-culture demonstrated that α-syn deposited in astrocytes is transferred to microglia and degraded by microglia through direct contact *in vitro* [235]. On the other hand, excessive microglial phagocytosis of a-syn leads to the accumulation of a-syn in microglia, which induces microglia in an intense inflammatory state [171]. Moreover, ineffective degradation of a-syn in microglia may lead to the release of a-syn through extracellular vesicles and exacerbate the spread between cells [236]. The extracellular oligomer a-syn can also exacerbate synaptic dysfunction and neuronal loss by affecting the phagocytosis and autophagy of microglia. For example, α-syn can inhibit microglia phagocytosis by blocking FcγR signaling [237]. An *in vitro* study demonstrated that extracellular α-syn inhibited the initiation of microglia autophagy by activating TLR4 and Akt-mTOR signaling [238]. An *in vivo* study has shown that deletion of microglia autophagy-associated gene 5 (ATG5) exacerbated the loss of dopamine neurons in mice [239].

As the disease progresses, progressive loss of synapses becomes the most important pathological feature and leads directly to motor deficits [5]. Synaptic loss is the result of multiple factors, including presynaptic dysfunction due to the accumulation of a-syn [223], excitotoxicity due to pathological activation of NMDAR [240] and phagocytosis by reactive microglia in MPTP and 6-hydroxydopamine-induced PD mouse models, where direct contact of reactive microglia with dopamine neurons and phagocytosis of whole dopamine neurons was observed [241, 242]. Current studies suggest that phagocytosis of DA neurons by microglia may be mediated through the complement pathway or activation of TAM receptors. CR3 expression in microglia is increased in rotenone-exposed rats [243], and C3 knockdown rescues DA neurodegeneration in mice after injection of the bacterial component lipopolysaccharide (LPS) [244]. Microglia can also mediate phagocytosis of live DA neurons through upregulation of TAM receptors [245]; deletion of TAM receptors MER and AXL in α-syn transgenic mice increases the lifespan of mice [58].

Another non-negligible role of microglia in PD is to delay the onset of motor symptoms as a compensatory mechanism. Approximately 30-60% of dopaminergic neurons are lost by the time PD patients show movement disorders [246]. Dopinamergic is transmitted downstream motor neurons *via* the striatum, which mainly consists of GABAergic spiny projection neurons (SPNs). Spiny projection neurons receive DA innervation from the substantia nigra pars compacta and glutamatergic inputs from the cerebral cortex and thalamus. They are divided into two subgroups: some direct project input signals to the substantia nigra pars reticulata and the globus pallidus (direct pathway dSPNs); the other part projects to the external segment of the globus pallidus (GPe), which is connected to the subthalamic nucleus (STN) (indirect pathway iSPNs) through GABAergic synapses, and the STN releases glutamate. The progressive loss of DA inhibits dSPNs and activates iSPNs or STN. The activated STNs release excess glutamate to excite substantia nigra pars reticulata/globus pallidus, which exerts inhibitory effects on downstream motor regions through GABAergic and inhibits movement [247]. Moreover, excessive accumulation of glutamate at synapses leads to Ca^2+^ overload and apoptosis, which further promotes the loss of DA neurons [240]. However, glutamatergic synaptic hyperactivity is regulated by microglia. Activated microglia inhibit glutamate cytotoxicity by phagocytosis of overactive glutamatergic synapses in the substantia nigra pars reticulata and the globus pallidus of a 6-hydroxyapto-induced rat *Hemiparkin-Sonism* model [248]. Activated microglia also promote extrasynaptic glutamate reuptake by expressing excitatory amino acid transporter-2 (EAAT-2) [249]. This suppresses overexcited glutamatergic synapses which leads to the delayed appearance of motor symptoms, despite the massive loss of DA neurons.

#### 5.2.3 MS

MS is a demyelinating disease of the CNS mediated by inflammation and immunity, which can involve the brain, spinal cord, and optic nerves. The etiology of MS is unclear, and it is related to genetic and environmental factors [250]. The pathological process of MS involves demyelination. Therefore, it was thought that only white matter is affected in MS. However, further studies found that gray matter is also affected in the early stages of MS and is directly related to cognitive dysfunction and long-term disability of patients [251]. Gray matter damage in MS patients includes demyelination, synaptic damage, and neurodegeneration [252].

Unlike other neurodegenerative diseases, MS is characterized by a breakdown of BBB and the introduction of peripheral immune cells (T cells, B cells, and macrophages) into the CNS [253]. Microglia are the resident immune cells of the CNS and begin to accumulate in the early stage of MS. This is known as the “microglia nodule”, which is regarded as an early sign of MS [172, 254]. To determine the role of microglia in MS, Martina *et al.* analyzed the edges of demyelinating white matter in frozen human brain tissue by single-nucleus RNA-sequencing analysis and identified and defined MIMS. MIMS has two clusters with different functions. In the first MIMS cluster, pathway analysis enriched for lysosome, response to lipoprotein particles, and regulation of inflammatory response, which is consistent with the phagocytosis and clearance of myelin by microglia. The second MIMS cluster had higher expression of MHC-associated and inflammatory markers, including SOD1, iron-related genes, and complement C1-complex genes, consistent with an antigen presentation and pro-inflammatory role for microglia [55]. Microglia in a pro-inflammatory state can express pro-inflammatory cytokines (such as TNF-α, IL-1, C1q), ROS, and glutamate, which promote oxidative stress and directly lead to myelin damage. Meanwhile, it induces a toxic phenotype of astrocytes and damages oligodendrocytes, which can produce myelin [252]. The release of glutamate is mainly ascribed to the opening of microglia HCs, and the application of the gap junction blocker carbenoxolone (CBX) has been shown to attenuate symptoms in EAE mice by reducing glutamate release by reactive microglia [255]. In addition, microglia can induce their T cell inflammatory response by the antigen presentation function to assist the spread of inflammation in the brain [252, 253]. The phagocytosis function of microglia is critical in MS and is strongly dependent on the innate immune receptor TREM2. Microglia can remove defunct myelin debris by phagocytosis, which contributes to myelin regeneration. Microglia in gray matter can even participate in iron processing: removal of iron that exacerbates oxidative damage and promotes iron uptake (necessary for myelin synthesis) by oligodendrocytes. In addition, microglia can phagocytose apoptotic cells and prevent their further release of inflammatory factors [252, 253, 256].

Synapses are abundant in gray matter; therefore, their structural and functional impairments are directly related to cognitive dysfunction in MS [257]. The brain pathology of MS patients shows decreased synaptic density, impaired synaptic transmission function, and disorder of neurotransmitters. The main “murderers” of these adverse consequences are thought to be microglia [252]. Proinflammatory factors (such as TNF-α and IL-1) and ROS produced by MS microglia directly damage the LTP of synapses [258], affect the glutamate reuptake of astrocytes, and lead to increased neurotoxicity and damaged synaptic function [257, 259]. In addition, microglia can affect the survival of synapses through phagocytosis mediated by complement (C3 and C1q). Current studies prove that the contents of the colocalization of complement cascade components and synaptic proteins in the MS model are negatively correlated [260-262]. The removal of apoptotic synaptic material can block the spread of harmful substances, thereby protecting the surviving synapses, and indirectly playing an anti-inflammatory role by inhibiting the spread of inflammation [252].

### 5.3 Stroke

#### 5.3.1 Ischemic Stroke

Ischemic stroke is one of the most common diseases threatening human health. Microglia are one of the first immune cells activated after ischemic stroke. Microglia activation is detected in the infarct core 24-48 h after ischemic stroke in humans and expands into the peri-infarct region over time. This activated state can last up to 14 weeks [263]. Experimental stroke involves activation of microglia within minutes of cerebral ischemia, with a peak at 48-72 h that persists for several weeks [264, 265].

##### 5.3.1.1 Roles of Microglia on Synapses in the Acute Phase of Ischemic Stroke

In the hyperacute phase of ischemic stroke, microglia are located in the ischemic core and gradually accumulate around the infarct [266]. A study identified 6 microglia clusters in the ischemic penumbra of mice 24 hours after MCAO by using single-cell RNA sequencing. Subsequent gene set variation analysis (GSVA) revealed that four of these clusters were characterized by enriched hypoxia, TNF-α, IL-6, and IL-2 inflammation-related genes and pathways, indicating that microglia presented an intense inflammatory state during this period [173]. Another single-cell sequencing of the ischemic cerebral hemisphere in mice 24 hours after MCAO also revealed similar results. Five microglia clusters (MGs) were identified, of which MG2, MG3, and MG4 were highly associated with ischemic brain injury, showing high expression of genes related to inflammation, matrix metalloenzyme MMP12 and proliferation [267]. Overall, microglia showed a pro-inflammatory and highly proliferative state 24 hours after MCAO. And the state continued till day 3 after MCAO. ScRNA-seq analysis of aged brains at this time point has revealed 6 MGs, with stroke-associated MG5 and MG6 highly expressing genes related to proliferation-related and pro-inflammatory signaling (CXCR2, S100a8, Il-1β, and MMP9), respectively [268]. Hence, microglia are primarily in a pro-inflammatory state during the acute phase of ischemic stroke, aggravating nerve damage through phagocytosis and release of pro-inflammatory cytokines (IL-1β, IL-6, and TNF-α), ROS, proteases, chemokines and other inflammatory mediators. Damaged neurons release “Find Me” signals such as ATP, uridine triphosphate, and LPC3 to induce the recruitment of microglia [265]. The recognition of PS on the surface of neurons causes reactive microglia to phagocytose the surviving neurons in the penumbra and aggravate the functional deficits [269]. In addition, the capture and phagocytosis of endothelial cells by reactive microglia increases the blood-brain barrier (BBB) permeability of the aggravating brain edema [270]. Proinflammatory cytokines IL-1β and TNF-α promote the expression of chemokines such as CINC to increase the infiltration of peripheral leukocytes and directly increase BBB permeability, thereby aggravating angioedema and post-hemorrhage transformation [271]. What’s more, Reactive oxygen species and MMP9 directly lead to BBB destruction through oxidative stress and ECM degradation, respectively [272]. The reactive microglia also up-regulate the expression of chemokines such as monocyte chemoattractant protein-1 (MCP-1), CCL5, and CXCL8 to induce infiltration of peripheral leukocytes and further aggravate neuroinflammation [273]. Local severe inflammatory responses lead to apoptosis of oligodendrocytes, followed by axon demyelination [274].

Nevertheless, the neuroprotective effect of reactive microglia during this period cannot be ignored despite their relatively small number. Microglia engulf dead cells or synaptic debris and neutrophils located around the infarction, thereby alleviating local inflammatory damage [266, 275]. Furthermore, microglia, the ECM, and reactive astrocytes are the main components of glial scars, which form in the periinfarct area 3-5 days after ischemic stroke [276]. Ym1 released by microglia prevents ECM degradation, thereby promoting glial scar formation [277]. Glial scars seal off the damaged area and prevent the spread of inflammation and cellular damage. They can also stimulate the reestablishment of capillaries and provide nutritional support to surviving nerve tissue [278].

##### 5.3.1.2 Roles of Microglia on Synapses in the Chronic Phase of Ischemic Stroke

The generation of an early series of inflammatory injuries causes the brain to begin to “save itself” through neurogenesis, angiogenesis, formation of new synapses, regeneration of myelin, and reconstruction of neurovascular units [279]. Seven days to three months after an ischemic stroke is a critical period of nerve regeneration and synaptic remodeling [280]. The transition of microglia from the pro-inflammatory state to the anti-inflammatory state is crucial for neural recovery [281]. Microglia in this phase are more branched or intermediate in morphology [282]. Functionally, microglia exhibit phagocytosis of necrotic tissue debris and anti-inflammatory and neurotrophic effects. The anti-inflammatory factors IL10 and TGF-β produced by microglia help reduce oxidative stress and promote neurogenesis [283]. Neurotrophic factors such as IGF1 released by microglia promote nerve regeneration in the subventricular zone after ischemic stroke [10]. Microglia also promote myelin sheath regeneration by releasing activin-A [284]. Furthermore, microglia produce nutrient gradients that guide the growth of new nerve fibers along the infarct margin [285]. Microglia also promote synaptic remodeling by releasing MIR124-rich exosomes, which inhibit astrocyte proliferation and reduce glial scar formation [286]. Moreover, SPARC released by microglia is beneficial to enhance synaptic strength and reduce hypoxic-induced nerve damage. Neurons pretreated with recombinant SPARC in oxygen-glucose deprivation (OGD) conditions show reduced synaptic loss [287]. Glial scarring around the infarct during this period is more pronounced and firmed, with blocking of the nerve and synaptic regeneration around the infarct limiting the recovery of neural function in the chronic phase, unlike in the acute phase [276]. Microglia phagocytose surviving neurons and synapses (including excitatory and inhibitory synapses) in this area by up-regulating MEGF10 and MERTK, which further aggravates neurological dysfunction [288].

**Fig. (4):**
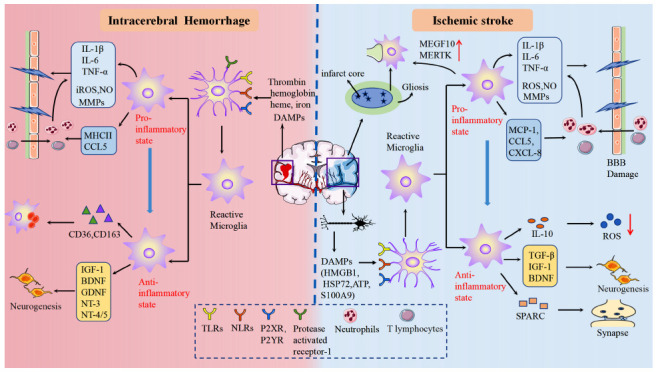
Microglia-synapse interactions in stroke. Dead neurons and blood components induce reactive microglia after stroke. Microglia are primarily in a pro-inflammatory state during the acute phase of stroke. Reactive microglia aggravate neuronal death, BBB disruption, and brain edema by releasing pro-inflammatory factors, peroxides, MMPs, and chemokines. Microglia gradually transform into a pro-inflammatory state as the disease progresses. Microglia phagocytose synaptic debris and release IL-10, TGF-β, IGF-1, and BDNF in ischemic stroke to suppress inflammatory responses and promote neurogenesis and synaptic remodeling. Microglia promote hematoma clearance mainly in intracerebral hemorrhage by up-regulating the expression of phagocytic receptors CD36 and CD163 and expressing a large number of nerve growth factors to promote synaptic remodeling.

In conclusion, microglia exhibit a diversity of functions and states in different phases after ischemic stroke. The pro-inflammatory state of mouse microglia during the acute phase of ischemic stroke was revealed by applying single-cell RNA sequencing, which is currently one of the most accurate methods to reflect the transcriptional characteristics of microglia. However, the current single-cell RNA sequencing analysis of microglia is still limited to the acute phase of MCAO in mice and cannot reveal the continuous changes of microglia after ischemic stroke. It is necessary to analyze the transcriptional characteristics of microglia in different phases of human stroke (Fig. **4**).

#### 5.3.2 Intracerebral Hemorrhage (ICH)

Intracerebral hemorrhage is the second most common type of stroke, accounting for 10-15% of all stroke cases [289, 290]. The mortality and disability rates of ICH remain high due to the lack of effective treatment [291]. Brain injury caused by ICH includes primary brain injury and secondary brain injury. Primary brain injury is the mechanical destruction of surrounding brain tissue caused by a rapidly forming hematoma [289]. Secondary brain injury is mainly attributed to the extravasation of blood components, which activates excitotoxicity, oxidative stress, and inflammatory pathways, including microglia and peripheral immune cells. This results in BBB destruction, progressive brain edema, hematoma enlargement, and worsening nerve damage [292]. Microglia are the first immune cells to respond to brain damage. Microglial response as early as 1 hour after ICH peaks within 1-3 days and returns to normal after several weeks in mouse models of ICH [293].

##### 5.3.2.1 Roles of Microglia on Synapses in the Acute Phase of ICH

Microglia respond to blood components, including thrombin, hemoglobin, heme, iron, and DAMPs released by dying neurons to be reactive by activating TLRs and protease-activated receptor-1 [294-296]. The reactive microglia around the hematoma are mainly in the proinflammatory state within 1 week after intracerebral hemorrhage [297]. Reactive microglia initially play a brief role in phagocytosis to remove the dead nerve cells and red blood cell debris [298]. However, the accumulation of reactive microglia gradually mediates neurotoxicity mainly through pro-inflammatory effects, leading to axonal degeneration around the hematoma, dendritic spine atrophy, and synaptic density reduction [299]. Microglia are the main source of proinflammatory mediators such as IL-1β, TNF-α, and IL-23, which aggravate local inflammation [300]. Microglia also release high levels of glutamate, and ATP attributed to the activation of Cx43 HCs [301], leading to the opening of Panx1 channels and Cx36 hemichannels in neurons. The ensuing neuronal Ca^2+^ overload further aggravates neuronal injury [302]. Furthermore, MHCII overexpressed by reactive microglia can induce T-cell infiltration through antigen presentation and aggravate tissue damage [270].

##### 5.3.2.2 Roles of Microglia on Synapses in the Chronic Phase of ICH

The intracranial inflammatory microenvironment is transformed during ICH. Anti-inflammatory factors such as IL-4, IL10, and TGF-β are significantly increased around the hematoma, which promotes the transformation of microglia from the pro-inflammatory state to the anti-inflammatory state [297, 303]. Indeed, mRNA levels of markers of anti-inflammatory phenotypes, such as Arg-1, Ym1, CD206, and IL-4, increase within 1-3 days in collagenase-induced ICH models [304]. Reactive microglia are primarily in the anti-inflammatory state within 1-2 weeks after ICH [305], which are involved in tissue repair and neurological function recovery in the late stage of ICH. First, Microglia engulf hematoma and dead tissue to create a favorable environment for tissue regeneration. CD36 and CD163 are phagocytic receptors expressed by microglia. CD36 belongs to the class B scavenger receptor family and plays an important role in the clearance of hematoma. Hematoma absorption is significantly inhibited after intracerebral hemorrhage in CD36 knockout mice and is accompanied by the aggravation of neurological deficits [306]. CD163 also promotes hematoma clearance by absorbing hemoglobin [307]. In addition, various trophic factors and growth factors produced by microglia, including IGF1, BDNF, GDNF, and neurotrophin such as NT-3 and NT-4/5, are involved in the regulation of nerve regeneration, axonal growth, and synaptic remodeling and promote the recovery of brain structure and function [11] (Fig. **4**).

### 5.4 TBI

TBI is the injury of the brain caused by external mechanical forces, which leads to neurological dysfunction and mental disorders [308]. Traumatic brain injury patients are at significantly increased risk of cognitive decline and psychiatric comorbidities compared with the general population [309]. Current TBI treatments are limited since it is a heterogeneous disease; the different nature (including brain contusions, concussions, and blast injuries) and severity of trauma determine the different pathological mechanisms and clinical consequences of the disease. Currently, no animal model can simulate all types of human TBI. Some treatments that show promise in preclinical studies have repeatedly failed in clinical trials [310]. Meanwhile, a biochemical cascade called “secondary brain injury” is triggered after mechanical injury, which starts within minutes after injury and lasts for months [311]. This leads to the loss of neurons and synapses, extensive axonal damage, and impaired synaptic plasticity [312]. Secondary brain injury is mediated by multiple pathways at the cellular level, including glutamate-mediated excitotoxicity, oxidative stress, and local or systemic inflammation [313].

Reactive microglia can be observed after at least 72 h in human brains [314] and within 24 h in mouse brain injury models [315]. Reactive microglia play dual roles in the acute (48 h) and subacute (7 d) phases of brain injury in mice. Reactive “satellite” microglia encase and engulf CA1 excitatory synapses and cause cognitive impairment in mice within 1 week after focal injury in a mouse model of focal brain injury [174]. Microglia produce excessive inflammatory mediators such as IL-1β, IL-6, TNF-α, Mir-155, MMPs, NO, and ROS, which can aggravate the inflammatory response by increasing the permeability of the BBB and promote the infiltration of peripheral immune cells [316, 317]. In addition, the mice chronic constriction injury (CCI) model demonstrated that microglia promote peripheral leukocyte infiltration through activation of Panx1 channels, whereas Panx1 gene deletion reduced leukocyte infiltration and decreased post-TBI memory and locomotor dysfunction [318]. However, microglia during this period also have neuroprotective effects. For example, damaged astrocytes attract a large phagocytic network of microglia, which are involved in removing cellular debris and maintaining the integrity of the glial limit protein barrier [319]. Depletion of microglia immediately after closed brain injury in neonatal rats does not provide any benefit and leads to increased neurodegeneration [320]. Exosomes enriched with Mir-124-3p produced by microglia peak at 7 days after brain injury and attenuate neurodegeneration in injured neurons *in vitro* and *in vivo* [321].

Microglia persist in a reactive state in the injured brain after the acute inflammation subsides. The mouse TBI model demonstrates that microglia can persist reactive for months [322]. Microglia remain reactive for years after TBI in humans [323]. This mediates chronic inflammation in the brain and leads to long-term neurological dysfunction. Amoebic reactive microglia in the acute phase (1-2d) and chronic phase (28-35 d) significantly increase in the ipsilateral cortex, thalamus, and hippocampus using the mouse CCI model. At the same time, the mice showed significant cognitive and learning deficits [324, 325]. Microglia also mediate cognitive impairment by phagocytosis of neurons and affect synaptic plasticity. There is continuous complement activation in the brain of mice within a few months after TBI. The deposition of C3 and C1q in hippocampal synapses mediates their phagocytosis by microglia, which leads to the loss of hippocampal synapses and the decline of cognitive function in mice [326]. Meanwhile, inhibition of the complement pathway (using the inhibitor CR2Crry) improves the synaptic density and cognitive performance of mice [327]. Furthermore, microglial genes related to LTP (such as *PTPN5 and Sqstm1*) are gradually upregulated 7-90 days after TBI, and these genes are associated with other neurocognitive disorders, including AD, schizophrenia and autism [328].

Chronic inflammatory damage mediated by microglia is associated with age. Older mice have worse functional outcomes after TBI than younger mice. This is partly because reactive microglia in aged mice exhibit limited protective effects such as reduced phagocytic activity and exercise capacity and greater toxicity (pro-inflammatory features) [329]. Some studies focused on the depletion of microglia in the chronic phase of TBI have shown therapeutic benefits for improving the neurological function of microglia. Mice treatment with CSF1R inhibitor Plexxikon5622 1 month after CCI to remove activated microglia, followed by deactivation of the inhibitor one week later, allows microglia regeneration, which significantly improves the long-term cognitive and motor impairment [330]. This may benefit from the neuroprotective effect of regenerated microglia. Morphologically, the regenerated microglia exhibit a bifurcated, nearly quiescent morphology. Regenerated microglia exhibit different genetic properties from chronically activated microglia at the transcriptional level. There is substantial downregulation of type I interferon pathway components (such as *IRF7*, *IFIT3,* and *MX1*) and upregulation of genes involved in wound repair (such as *FN1*, *ALCAM,* and *CSPG4*) [331]. Therefore, regenerated microglia may contribute to the recovery of long-term motor and cognitive function by reducing inflammation and neuronal death and promoting the regeneration of hippocampal nerves.

### 5.5 Epilepsy

Epilepsy is one of the most common neurological diseases and affects 50 million people worldwide [332]. It is characterized by hyperexcitability and abnormal synchronized excess firings of neuronal populations [6]. It is generally believed that the main pathogenesis of epilepsy is caused by the imbalance between excitatory and inhibitory neurotransmitters; the excitatory neurotransmitters transmitted by glutamatergic signals are increased while the inhibitory effect mediated by GABA_A_ receptors is impaired [333]. However, neuronal dysfunction and injury cannot fully explain the cause of epilepsy. Microglia in the CNS also play a significant role in the seizure and development of epilepsy.

Microglia can participate in the pathophysiology of epilepsy, including affecting neuronal function through inflammatory- and mTOR-mediated non-inflammatory pathways [334]. Microglia are activated by ATP, glutamate, and ROS released by damaged neurons. Reactive microglia begin to produce pro-inflammatory mediators within 30 min after a seizure. This occurs before the changes in cell morphology, and the expression level of pro-inflammatory mediators is positively correlated with the number and frequency of seizures. Activated microglia work together with astrocytes to release cytokines (including TNF-α), which reduce glutamate uptake by astrocytes and promote glutamate release by microglia and astrocytes, thereby increasing excitotoxicity of postsynaptic neurons and reducing the presence of GABA_A_ receptors. It reduces the inhibitory effect of synaptic excitation and further induces synaptic dysfunction and seizures [6]. At the same time, elevated *TGFβ1* mRNA is found in the hippocampus and temporal lobe of the rat epilepsy-induced model. TGFβ1 is believed to inhibit the inflammatory effect of microglia. In addition, some anti-inflammatory cytokines in microglia (such as IL-4 and IL-10) are detected in the epilepsy model; therefore, the pro-inflammatory or anti-inflammatory phenotype of microglia after epilepsy remains elusive to a large extent [6, 335]. Increased *mTOR* expression in microglia is found in the brains of patients with epilepsy; this is related to epileptogenicity and does not involve inflammatory changes [336]. mTOR affects epilepsy by changing microglia. First of all, the increase in mTOR changes the morphology of microglia, and selectively elevated mTOR in mice models of epilepsy contributes to microglia transitioning to amoeba morphology, thereby increasing microglia contact with neurons and astrocytes. Elevated mTOR signaling also increases microglia proliferation, lysosomal gene expression, and microglia phagocytosis function which enhances the “surveillance” function of microglia [334].

The increase of complement molecules C1q and C3 is found in microglia in the hippocampus of epileptic patients and epileptic model rats [337, 338], stimulating microglia to phagocytose synapses. A similar situation occurs for CX3CL1 secreted by neurons and CX3CR1 secreted by microglia in epilepsy models [6, 339]. All C1q-positive neurons in normal aging are GABAergic neurons; therefore, it was hypothesized that C1q released by microglia first accumulates in GABAergic neurons, thereby contributing to the loss of inhibitory neurons [339, 340]. In addition, neurons produce more ATP when synapses are overactive in the epileptic state, a phenomenon that can occur in excitatory and inhibitory neurons. Adenosine triphosphate can either be used as a targeted recognition signal of microglia or may be related to the extension of microglia branches, thereby increasing the contact between microglia and neurons and promoting the phagocytosis of microglia to synapses [339].

## 6 CLINICAL APPLICATION OF MICROGLIA INHIBITORS IN CNS DISEASES

Microglia are activated and change function and phenotype after CNS disease. Although reactive microglia have a certain protective effect on the CNS, long-term and uncontrolled activation may further aggravate the neurodegeneration process and neurological dysfunction. This was further explored through the use of microglia inhibitors to observe changes in neurological function in disease models.

A number of drugs may exert therapeutic effects *via* microglia in AD. For example, VX-745 is currently in Phase 2 of clinical trials and has been shown to act as a P38 MAPK inhibitor that can effectively relieve the progression of AD. It is an oral drug with selective p38α blocking activity, and p38 is involved in microglial response and pro-inflammatory cytokine release [341, 342]. The addition of minocycline (a tetracycline derivative) to glutamate-exposed cells showed that it inhibits the proliferation of microglia and the response of microglia through the p38 MAPK pathway. It also reduces the production of pro-inflammatory factors such as IL-1β, TNF-α, and NO, thereby protecting nerve cells [343]. In animal models, this drug has shown the potential to inhibit microglial response, reduce Aβ deposition, and inhibit neurodegeneration [344]. Pioglitazone acts as a PPATγ agonist to help microglia shift from a pro-inflammatory to an anti-inflammatory state, decreases the release of pro-inflammatory factors, and increases the phagocytosis of Aβ by microglia [345]. A specific CSF1R inhibitor, PLX5622, which provides sustained and long-term elimination of microglia, was found to inhibit plaque deposition in the mouse model of AD, thereby inhibiting the progression of disease [346]. In addition, since microglia have immune properties, inhibiting microglia response in the pre-disease phase may be helpful in the treatment and prevention of the disease [347].

Because of the immune cell properties of microglia, drug therapy in PD is mainly directed at reducing microglia-mediated neuroinflammation and neurological damage by maintaining the inflammatory response of microglia and reducing the conversion of microglia to the disease states. Amantadine, a commonly used drug, has also been shown to inhibit the response of microglia, thereby inhibiting neuronal damage caused by disease-associated states of microglia. Amantadine can also inhibit the production of pro-inflammatory factors by inhibiting the NF-kB pathway in microglia [348]. In addition, tanshinone and α-asarone can also inhibit the pro-inflammatory effects of microglia by inhibiting NF-kB [349]. The Drug candidates, including JNJ7777120 and MCC950, can be targeted and inhibit the NLRP3 inflammasome, thereby reducing the release of inflammatory factors from microglia [350, 351]. Drug candidates such as pioglitazone and rosiglitazone target peroxisome proliferator-activated receptor gamma (PPAR-γ) to inhibit the pro-inflammatory effects of microglia [352, 353]. Pyrroloquinoline quinone (a naturally occurring redox cofactor) inhibits the release of NO and pro-inflammatory factors IL-1β, IL-6, and TNF-α from microglia and activates the typical mitochondrial autophagy pathway (PINK1/Parkin pathway), which enhances autophagy of damaged microglia, thus exerting a protective effect against microglia-mediated cell death [354].

Disease Modifying Therapies (DMTs) are the primary treatment of MS. In recent years, the role of Bruton Tyrosine Kinase (BTK) Inhibitors (such as ibrutinib) in MS has been gradually explored. BTK belongs to the TEC family of non-receptor tyrosine kinases and has been proven to be expressed in B cells as well as myeloid cells, including monocytes, macrophages, microglia, mast cells, basophils, and neutrophils [355]. In patients with secondary progressive multiple sclerosis (SPMS) or relapse-remitting multiple sclerosis (RRMS), BTK activation was found to be significantly increased in B cells compared with healthy controls [356], suggesting that BTK inhibitors may have great therapeutic potential for MS. Notably, BTK expression in microglia has been identified in human brain tissue, and single-cell RNA sequencing shows that both BTK mRNA and BTK activation signals are highly abundant in microglia from patients with MS (only shown in the abstract of the literature) [357]. BTK inhibitors can modulate adaptive and innate immunity in the pathological process of MS by directly regulating the function of B cells and myeloid cells [358]. Although there is currently no evidence of a direct effect of BTK inhibitors on T cells, it has been suggested that BTK inhibitors may protect the CNS by affecting adverse B cell-T cell interactions [359]. Notably, BTK is expressed in microglia in human brain tissues, and single-cell RNA sequencing showed that both BTK mRNA and BTK activation signals were highly abundant in microglia from MS patients [357]. Current evidence suggests that BTK inhibitors target microglia to alleviate MS in several ways: **①** BTK inhibitor reduces the level of pro-inflammatory factors and antigen presentation of microglia by reducing the response of microglia [355]. **②** BTK inhibitors reduce the recruitment of microglia to immune cells by altering their gene expression, including genes involved in proinflammatory cytokines and chemokines [360]. **③** BTK inhibitor may promote myelin repair through microglia: Although there is no direct evidence that the effect of BTK inhibitors in promoting myelin repair is realized through microglia, the researchers found that microglia accounted for 75% of the cells expressing BTK and p-BTK. Therefore, microglia may be involved in the mechanism [357]. In addition, siponimod, a highly selective sphingosine 1-phosphate (S1P) receptor modulator, is thought to inhibit the pro-inflammatory states of microglia and reduce the release of inflammatory factors [361]; CNS can also get beneficial effects from siponimod by shifting microglia to a regenerative state [362]. Dimethyl fumarate (DMF) is currently approved for the treatment of adults with relapsing forms of multiple sclerosis. DMF promotes the attenuation of neuroinflammation by activating the antioxidant effects of NRF2 on microglia and reduces the expression of IL1β and TNF-α; DMF also regulates GSH to enhance microglial antioxidant capacity, maintains microglial iron homeostasis, and promotes anti-inflammatory microglial states, thereby promoting myelin formation [354]; DMF can down-regulates the transcription of purinergic receptors P2Y12 and P2Y6 in the pro-inflammatory phenotype, and reduces ATP-induced microglial reactivity, thereby inducing microglial protection for CNS [363].

CSF1R inhibitors are protective, whether in ischemic stroke or ICH. The administration of Ki20227 in stroke model mice reduces the infarct volume, significantly improves the neurobehavioral defects, decreases the expression of pro-inflammatory factor (TNF-α) in the infarct site, and increases the expression of anti-inflammatory factor IL-10 compared with the control group [364]. In a mouse model of aged ICH, regenerated microglia attenuated neuroinflammation, neurological deficits, and cerebral edema when PLX3397 was given to eliminate microglia and discontinued before hemorrhage induction [365]. CSF1R inhibitor also has a protective effect on the nervous system after TBI. Henry, R.J. The addition of PLX3397 in a mouse brain injury model causes short-term elimination of microglia to reduce chronic inflammation after TBI, which improves neurological function and the sensory-, motor-, and cognitive function in mice 3 months after injury [366]. In addition, melatonin treatment is thought to ameliorate neurological injury in mice by promoting microglia to anti-inflammatory states in a STAT3-dependent manner [367].

Sirolimus (rapamycin) belongs to the group of mTOR inhibitors, which can prevent seizures or premature death when given before or after a seizure and can have an antiepileptic effect after TBI by decreasing microglia response in a mouse model of TBI [368]. Minocycline given for 14 continuous days after status epilepticus in adult animals inhibits microglia response and nerve loss, reduces inflammatory factors, and thus reduces the frequency, duration, and severity of recurrent seizures [368, 369] (Table **2**).

In general, the protective effect of microglial inhibitory drugs on the CNS was confirmed in the laboratory, and these drugs have strong clinical relevance. They will play a powerful role in the treatment of human CNS diseases.

## CONCLUSION AND FUTURE PERSPECTIVES

Microglia, being one of the immune cells of the brain, highly regulate synaptogenesis, synaptic pruning, and synaptic plasticity under physiological conditions underlying the refinement and functioning of neural circuits. Meanwhile, in various CNS disorders, reactive microglia are involved in the progression of diseases by promoting inflammatory responses and oxidative stress as well as phagocytosis to regulate synaptic pruning, synaptic plasticity, and neuronal damage and repair (Table **1**) [370-375]. In this paper, we discuss the known evidence of the role of microglia on synapses in several neurological disorders, which may potentially provide new targets for the therapy of these diseases. In addition, the high heterogeneity of microglia makes it possible to precisely target microglia in specific functional states for the therapy of CNS diseases. In this era of multi-omics, there is no consensus on the definition of microglia heterogeneity. The insight into microglia heterogeneity at the transcriptional level by single-cell sequencing has generated huge interest, mainly due to the inference of functional heterogeneity by transcriptional features. The application of single-cell sequencing technology in AD, PD, MS, and ischemic stroke has identified several disease-associated microglia clusters. However, future work is still desired to elucidate the functional heterogeneity of these microglia in different transcriptional states.

## Figures and Tables

**Table 1:** The role of microglia on synapses in CNS diseases.

**Disease**	**Synaptic Defect**	**Involvement of Microglia**	**References**
**Alzheimer’s disease**	1. NMDAR hyperactivation in the postsynaptic membrane. 2. Loss of neurons and synapses.	1. Reactive microglia form a "barrier". 2. Microglia recognize and phagocytose damaged synapses. 3. Abnormal synaptic pruning: Microglia mediate excessive synaptic pruning through the complement pathway and the Sirpα-CD47 signaling axis leading to synaptic loss. 4. Reactive microglia continuously express pro-inflammatory and neurotoxic factors, and aggravate the damage of synapses.	[176, 178, 190, 194, 195, 205, 206, 213]
**Parkinson’s disease**	1. Blocked presynaptic vesicle fusion and DA release. 2. Loss of striatal DA neurons and synapses.	1. Microglia inhibit the spread of α-syn and synapse damage by phagocytosing α-syn and through TLR4, TAM receptors and autophagic pathways. 2. Microglia promote the removal of extracellular α-syn and α-syn deposited in astrocytes. 3. Excessive microglial phagocytosis of α-syn leads to the accumulation of α-syn in microglia, which induces microglia in an intense inflammatory state and phagocytosis and autophagy dysfunction. 4. Reactive microglia mediate phagocytosis of DA neurons *via* the complement pathway and TAM receptor activation to exacerbate synaptic loss. 5. Microglia phagocytose glutamatergic synapses, promote glutamate reuptake, and delay the onset of motor symptoms.	[171, 232-239, 243-245]
**Ischemic stroke**	Decrease in synaptic density.	1. Microglia phagocytose surviving neurons and synapses around the infarct core by recognizing PS. 2. Microglia release pro-inflammatory factors, ROS, and MMP9 to promote neuronal and synaptic damage. 3. Microglia promote neurogenesis and synaptic remodeling by phagocytosis of synaptic debris and release of anti-inflammatory and neurotrophic factors.	[10, 266, 272, 285-288, 370-373]
**Intracerebral hemorrhage**	1. Axonal degeneration. 2. Dendritic spine atrophy. 3. Synaptic density reduction.	1. Microglia promote neuronal and synaptic damage by releasing IL-1β, TNF-α, IL-23, NO, and ROS. 2. Microglia phagocytose hematoma and synaptic debris by expressing the receptors, CD36 and CD163.	[11, 299, 300, 306, 307, 374]
**Traumatic brain injury**	1. Diffuse axonal injury. 2. Delayed axonal degeneration. 3. Loss of synaptic proteins and synapses. 4. Impaired synaptic plasticity. 5. Abnormal tau deposition at synapses.	1. Microglia wrap around damaged neurons and engulf synapses. 2. Microglia produce excessive pro-inflammatory factors and aggravate synaptic damage. 3. Continuously activated microglia mediate chronic inflammation, engulf synapses, and impair synaptic plasticity. 4. Microglia regenerated after chronic depletion promote synaptic remodeling and improve cognition	[174, 311, 312, 316, 317, 326, 328-330, 375]
**Multiple sclerosis**	1. Decrease of synaptic density. 2. Impairment of synaptic transmission function. 3. Disorder of neurotransmitters.	1. The pro-inflammatory factors and ROS produced by microglia directly damage the LTP of synapses. 2. Microglia affect the glutamate reuptake of astrocytes, and increase neurotoxicity and synaptic damage. 3. Microglia phagocytose apoptotic synapses and block the diffusion of harmful substances.	[252, 257-262]
**Epilepsy**	Imbalance between excitatory and inhibitory neurotransmitters.	1. Reactive microglia reduce glutamate uptake by astrocytes, promote glutamate release, and increase excitatory toxicity in postsynaptic neurons. 2. Microglia preferentially phagocytose GABAergic neurons in epilepsy.	[6, 333, 339, 340]

**Table 2:** Clinical application of microglia inhibitors in CNS diseases.

**Disease**	**Drug**	**Mechanism of Action**	**Effects on Microglia**	**References**
**Alzheimer’s disease**	VX-745	P38 MAPK inhibitor	Inhibits microglial responses and reduces the release of pro-inflammatory factors.	[341, 342]
	Minocycline	p38 MAPK pathway	1. Inhibits the proliferation of microglia. 2. Inhibits microglial responses and reduces the release of pro-inflammatory factors. 3. Reduce Aβ deposition.	[343, 344]
	Pioglitazone	PPATγ agonist	4. Make microglia shift from a pro-inflammatory to an anti-inflammatory state, decreases the release of pro-inflammatory factors. 5. Increases the phagocytosis of Aβ by microglia. 6. Make microglia shift from a pro-inflammatory to an anti-inflammatory state, decreases the release of pro-inflammatory factors.	[345]
	PLX5622	CSF1R inhibitor	1. Provides sustained and long-term elimination of microglia. 2. Inhibiting microglia response in the pre-disease phase.	[346]
**Parkinson’s disease**	Amantadine	NF-ĸB pathway inhibitor	3. Inhibits microglial responses and reduces the release of pro-inflammatory factors. 4. Inhibit disease-associated states of microglia.	[348]
	Tanshinone	NF-ĸB pathway inhibitor	Inhibit the pro-inflammatory effects of microglia.	[349]
	α-asarone	NF-ĸB pathway inhibitor	Inhibit the pro-inflammatory effects of microglia.	[349]
	MCC950	H4R antagonist	Inhibit the activation of microglia and reduce damage to dopaminergic neurons.	[350]
	JNJ7777120	NLRP3 antagonist	Inhibit the activation of microglia and reduce damage to dopaminergic neurons.	[351]
	Pioglitazone/ Rosiglitazone	PPAR-γ agonists	Inhibit the pro-inflammatory effects of microglia.	[352, 353]
	Pyrroloquinoline quinone	PINK1/Parkin pathway	1. Inhibits the release of NO and pro-inflammatory factors from microglia. 2. Enhances autophagy of damaged microglia through mitochondrial autophagy.	[354]
**Multiple sclerosis**	Tolebrutinib	Bruton Tyrosine Kinase (BTK) Inhibitors	3. Reduces the level of pro-inflammatory factors and antigen presentation of microglia by reducing the response of microglia. 4. Reduce the recruitment of microglia to immune cells by altering their gene expression, including genes involved in proinflammatory cytokines and chemokines. 5. Promote myelin repair through microglia.	[355, 357, 360]
	Siponimod	Sphingosine1-phosphate (S1P) receptor modulator	6. Inhibit the pro-inflammatory states of microglia. 7. Shifting microglia to a regenerative state.	[361, 362]
	Dimethyl fumarate	NRF2 antioxidant pathway /GSH modulation	8. Reduces the expression of pro-inflammatory factors. 9. Regulates GSH to enhance microglial antioxidant capacity. 10. Maintains microglial iron homeostasis. 11. Down-regulates the transcription of P2Y12 and P2Y6 to reduces ATP-induced microglial reactivity.	[354, 363]
**Ischemic stroke**	Ki20227	CSF1R inhibitor	Decreases the expression of pro-inflammatory factor (TNF-α) and increases the expression of anti-inflammatory factor IL-10.	[364]
	Melatonin	STAT3-dependent manner	Promote microglia to anti-inflammatory states.	[367]
**Intracerebral hemorrhage**	PLX3397	CSF1R inhibitor	Make microglia regenerate to attenuate neuroinflammation, neurological deficits and cerebral edema.	[365]
**Traumatic brain injury**	PLX3397	CSF1R inhibitor	Make short-term elimination of microglia to reduce chronic inflammation after TBI.	[366]
**Epilepsy**	Sirolimus (rapamycin)	mTOR inhibitors	Inhibit microglia response.	[368]
	Minocycline	p38 MAPK pathway	Inhibit microglia respons and reduces inflammatory factors.	[368, 369]

## LIST OF ABBREVIATIONS

AD: Alzheimer's Disease
CAM: Cell Adhesion Molecule
DA: Dopaminergic
ECM: Extracellular Matrix
EPSC: Excitatory Postsynaptic Currents
ICH: Intracerebral Hemorrhage
LTP: Long-term Potentiation
MS: Multiple Sclerosis
MV: Microvesicle
NMDR: N-methyl-D-aspartate Receptor
NP: Neuronal Pentapeptide
PD: Parkinson’s Disease
PS: Phosphatidylserine
SNr: Substantia Nigra Pars Reticulata
SPN: Spiny Projection Neuron
STN: Subthalamic Nucleus
TBI: Traumatic Brain Injury
